# Rationale for New York City’s Regulations on Nutrition, Physical Activity, and Screen Time in Early Child Care Centers

**DOI:** 10.5888/pcd11.130435

**Published:** 2014-10-16

**Authors:** Cathy Nonas, Lynn D. Silver, Laura Kettel Khan, Laura Leviton

**Affiliations:** Author Affiliations: Lynn D. Silver, Public Health Institute, Oakland, California; Laura Kettel Khan, Centers for Disease Control and Prevention, Atlanta, Georgia; Laura Leviton, Robert Wood Johnson Foundation, Princeton, New Jersey.

## Abstract

Childhood obesity is associated with health risks in childhood, and it increases the risk of adult obesity, which is associated with many chronic diseases. Therefore, implementing policies that may prevent obesity at young ages is important. In 2007, the New York City Department of Health and Mental Hygiene implemented new regulations for early childhood centers to increase physical activity, limit screen time, and provide healthful beverage offerings (ie, restrict sugar-sweetened beverages for all children, restrict whole milk for those older than 2 years, restrict juice to beverages that are 100% juice and limit serving of juice to only 6 ounces per day, and make water available and accessible at all times). This article explains why these amendments to the Health Code were created, how information about these changes was disseminated, and what training programs were used to help ensure implementation, particularly in high-need neighborhoods.

## Background: Why Act to Reduce Obesity in Early Childhood

In 2006, childhood obesity in the United States had reached record levels, even among children as young as 2 to 4 years (1,2). A New York City (NYC) Department of Health and Mental Hygiene (DOHMH) study of 16,000 children in NYC Head Start early childhood centers found that 27% of children were obese and 16% were overweight, or 4 of 10 preschoolers were at potentially harmful weights (3). It was clear that child obesity was associated with risk factors such as type 2 diabetes, high blood pressure, and nonalcoholic fatty liver disease among others (4) and that to address this issue public health interventions needed to begin when children are very young.

Health departments are required by law to protect and promote the health of their constituents. In the United States, 25% of all children aged 0 to 4 years are in a center-based child care program (5). There are approximately 2,000 such public and private group child care centers in NYC, caring for roughly 120,000 children aged 0 to 5 years. Unlike boards of health in other cities, the NYC Board of Health has long-held independent regulatory authority over group child care centers; therefore, it made sense for the DOHMH to prioritize early childhood settings for its initial obesity prevention interventions.

The DOHMH’s Bureau of Child Care has a team of inspectors and early childhood educators who examine each center at least annually and provide support to ensure compliance with applicable regulations. Until 2006, the Health Code included only general provisions relating to play and wholesome food that had been unenforced for some time. In 2006, the DOHMH proposed to the NYC Board of Health an amendment to Article 47 of the City’s Health Code to establish requirements for healthful beverages, strengthen requirements for physical activity, and limit television screen time provided to children. The proposal was introduced at the NYC Board of Health public hearing in March 2006, in conjunction with additional tuberculosis screening requirements at early child care centers. A public comment period was opened for 30 days after which an open hearing was held in April 2006. The proposal was uncontroversial. Only 4 parties commented, 3 who were supportive and 1 who raised the concern that the regulation could indirectly sanction television use. The final proposal was approved in June 2006 and became effective January 1, 2007. The new regulations were shaped by relevant national recommendations and guidelines. Some states had made changes in nutritional standards (6), but to our knowledge, this implementation of regulations was the first time a local regulatory authority addressed obesity in the early child care environment. Because these regulations applied to the entire population of city-licensed, group-based, early child care centers, these regulations have a broad reach. They also constituted a potentially low cost and sustainable approach to changing environments.

The articles in this issue of *Preventing Chronic Disease* present the results of an evaluation of the 2007 NYC group child care regulations conducted in 2010 by ICF International with funding from the Robert Wood Johnson Foundation and technical assistance from the Centers for Disease Control and Prevention (CDC), the NYC DOHMH, and New York University. As we seek to understand best practices for reducing childhood obesity and improving children’s health, several questions arise in relation to the NYC experience: were these regulations feasible to implement, are they effective at increasing physical activity or improving nutrition, and can they be replicated. This evaluation of the NYC regulations is a first step in better understanding best practices for child care centers. This introduction to the collection of manuscripts will seek to describe the content of and rationale for the regulations in NYC, and their evaluation will be discussed in the accompanying manuscripts (7–13).

## The Regulations and Their Rationale in 2006

### Nutrition

The regulation changes clarified requirements relating to beverages, with specific provisions including the following: 1) beverages with added sweeteners, whether artificial or natural, shall not be provided to children; 2) juice shall only be provided to children aged 8 months or older and shall not be provided in a bottle, only 100% juice shall be permitted, and children shall receive no more than 6 ounces per day; 3) when milk is provided, children aged 2 years or older shall be served milk with only 1% or less milk fat, unless milk with a higher fat content is medically required for an individual child, as documented by the child’s medical provider; and 4) water shall be made available and shall be easily accessible to children throughout the day, including at meals (Box).

**Box. Health code amendment to require healthy beverages, increase physical activity, and limit television viewing, New York City, 2007**

Physical Activity

- Aged 1–3 years: 60 minutes of physical activity per day.
- Aged 3 years or older: 60 minutes, including 30 minutes of guided and structured physical activity.

Television Viewing

- Younger than 2 years: restricted.
- Aged 2 years or older: 60 minutes or less per day.
- Only educational programs or programs that actively engage child movement (ask Early Childhood Consultants for ideas!)

Nutrition/Beverages

- No beverages with any added sweeteners, whether artificial or natural, shall be served.
  - Only 100% fruit juice is allowed — check Nutrition Facts Label.
  - No more than 6 ounces per day is allowed.
  - Juice shall only be provided to children 8 months or older and should not be provided in a bottle.
- Milk:
  - Only unsweetened/unflavored 1% or nonfat milk for children aged 2 or older is allowed. Milk substitutes (such as soymilk) must be unflavored and unsweetened.
  - Only unsweetened/unflavored whole milk for children ages 12 months to younger than age 2 is allowed.
- Water must be made available and easily accessible to children throughout the day (recommendation: directly on the table at meals and snacks).

By 2006, enough epidemiologic data identified sugary drink consumption as a factor in the obesity epidemic that ensuring that children do not consume these products routinely was considered a priority (14). There was also interest in acting early to reduce exposure to sweetened beverages; because infants have a preference for sweet taste, consuming sweetened beverages at an early age may perpetuate that preference throughout life (15). Sugar-sweetened beverages are energy dense and mostly nutrient poor, adding more calories and often unneeded nutrients to a child’s dietary intake. Since 2006, the body of literature on this issue has grown and the evidence base strengthened (16,17).

The fruit juice restriction was established because even among children aged 2 to 4 years, 100% fruit juice contributes roughly 100 calories to a daily diet (17,18). These beverage standards were consistent with recommendations from the American Academy of Pediatrics (19), as well as the 2005 Dietary Guidelines for Americans (20).

When the regulations were passed, there were no federal requirements in child feeding programs about the type of milk to be served. However, both the Dietary Guidelines for Americans and the American Heart Association with support from the American Academy of Pediatrics recommended low-fat or nonfat milk rather than whole milk for children older than 2 years. Since 2006, both the Special Supplemental Nutrition program for Women, Infants, and Children (WIC) (21) and the Child and Adult Care Food Program changed their requirements to serve low-fat milk for children older than 2 years (22). Additionally, restrictions on trans fat took effect in 2007, and a Mayoral Executive Order on standards for food purchased and served by all NYC agencies (including city-funded group child care centers under the Health Department’s jurisdiction) took effect in 2008. These food standards contained limits for sodium, an increase in fruits and vegetables, and an increase in fiber, as well as a restriction on added sugar and a reduction in fat. Many similar recommendations are supported by the Institute of Medicine (23,24).

Water, particularly tap water in NYC, is a low-cost, healthful way to keep children hydrated. The intent of the regulation requiring access to tap water was to accustom children to drinking it at an early age. The Healthy Hunger-Free Kids Act of 2010 also now requires that clean water be easily available in school (25), and studies have shown its potential for weight gain prevention (26). Therefore, a regulation that water be available and accessible at all times during the day in early child care centers seemed a simple and fundamental public health measure.

### Screen time

The updated regulations on screen time stated that

. . . television, video and other visual recordings shall not be used with children under two years of age. For children ages two and older, viewing of no more than 60 minutes per day of educational programs or programming that actively engages child movement. Children attending less than a full day program shall be limited to a proportionate amount of such viewing.

Television viewing is positively associated with an increase in body mass index (BMI). Evidence cited when the regulation was put into effect included the following: among children in the longitudinal Framingham Children’s Study those who watched the most television had the greatest increase in body fat from aged 4 to 11 years (27). A study of 3- to 4-year-olds found that the length of time spent in television viewing predicted BMI over 3 years and that this factor became an even stronger predictor over time (28). Evidence from the Institute of Medicine also indicated that food and beverage marketing targeted to children aged 12 years or younger led them to request and consume high-calorie, low-nutrient products (29).

Reducing television and screen exposure was a tenet of many health messages from such organizations as the Anthem Blue Cross/Blue Shield partnerships with Maine (30). However, most public health messages such as the 5-2-1-0 campaign (www.letsgo.org) call for 5 fruits and vegetables, no more than 2 hours per day of recreational screen time, 1 hour of physical activity, and 0 sugar-sweetened beverages per day. Since that interval includes time at home, it was felt that 60 minutes of screen time per full day at a child care center was a reasonable limit, and screen time content was restricted to educational programing or programming that promoted movement. Early childhood specialists from the Bureau of Child Care were available to specify appropriate programming.

### Physical activity

The Article 47 amendments established minimum standards for physical activity in early childhood. Specifically, full-day centers are required to provide children 12 months or older with 60 minutes of physical activity per day. For children aged 3 years or older, 30 of the 60 minutes each day must be structured. Evidence on the benefits of increased physical activity for young children was limited in 2006, although there were some studies on its effects on adiposity (31); there was much evidence on its importance for older children and adults (32), and it was believed that this activity level would be a precursor to healthful behaviors as children age. The proposed regulations cited the 2001 National Association for Sport and Physical Education recommendations that toddlers and preschoolers participate in at least 60 minutes of physical activity per day (33) and that young children not be sedentary for more than 60 minutes at a time except when sleeping. Therefore, establishing these minimum requirements for physical activity was in line with existing recommendations.

The new regulations had a clear statement that no matter how small the space or bad the weather physical activity had to programmed for the children, indoors or out. However, it was also clear that to help centers overcome some of the barriers to weather and space, substantial support and training would be needed. There were other reasons why physical activity training might be required: many NYC centers not only lacked space, but they lacked equipment; some staff members may not like to be physically active; staff members who are overweight or obese or have physical disabilities may find it difficult to perform certain activities; and studies indicate that children in high-poverty neighborhoods are less likely to have access to physical activity than other children (34).

## Implementing the Regulations

The Bureau of Child Care communicated the changes in regulations to child care centers through 3 channels. First, in late 2006, the Bureau invited all center directors to 1 of 5 public meetings organized in each of the 5 boroughs of New York City. Second, letters were sent to all centers about the new regulations in March 2007. Third, sanitarians and early childhood education consultants who annually visit the centers attended trainings about the regulations and were given directives to ensure that the center directors were aware of these changes. The early childhood education consultants are charged with supporting the centers’ education curriculum. The sanitarians are charged with documenting whether the centers are in compliance with all the regulations in the NYC Health Code. In 3 particularly high-poverty neighborhoods, additional on-site technical assistance related to nutrition and physical activity in general and the new regulations specifically was provided to centers from 2006 until 2010. This individualized technical assistance ended after all sites in each of these neighborhoods had been visited on at least 2 occasions each.

In 2006, NYC’s City Council funded expansion of a training program for staff in early child care centers to help them implement the new beverage and screen time regulations and the physical activity requirement in their classrooms. Since that time, approximately 14,000 teachers from over 70% of the 1,600 licensed centers that care for children 2 to 5 years of age have been trained. Manuals and play equipment were provided to centers that sent staff for training. At the time of this evaluation, most teachers had been trained through a program called Sports, Play, and Active Recreation for Kids! (SPARK!), part of Sportime, Inc (www.sparkpe.org/about-us/sportime/). The program had been edited to meet NYC needs for small space use. After this evaluation was complete, because of contractual issues, the DOHMH created its own physical activity curriculum called Move-To-Improve for Early Childhood, developed by DOHMH staff specifically for small spaces typical of NYC centers. The curriculum is publicly available at www.nyc.gov/html/doh/downloads/pdf/cdp/cdp-pan-staff-early-child-intro.pdf.

## Why a Regulatory Approach

One alternative to a regulatory approach is intensive education of and outreach to staff at child care centers. However, NYC, like many other jurisdictions, has large numbers of facilities and limited staff and funding. Although intensive technical assistance and outreach may have been effective in changing practices without regulation, the necessary resources to reach 2,000 facilities rapidly were simply not feasible without funding from outside sources.

In contrast, policy approaches, such as those used in NYC, can affect a large number of people and be instituted at a low cost for the population they reach. It would be naïve to assume that simply writing a regulation alone would automatically result in high levels of compliance with those policies. Effective communication of requirements, some level of technical support and training, and consistent enforcement are needed for most policies to be optimally successful.

Policy and program approaches also are not either–or. One could think of obesity policies as layers: each layer inches closer to better health and raises the tenor of the work. For example, in the case of reversing tobacco use, it has taken many layered policies — including smoke-free air in more places, taxation, hard-hitting media campaigns, and cessation support — over a decade to successively reduce the prevalence (35). In the field of obesity prevention, in 2006 whole milk was still part of school, early child care centers, WIC, and other places where children spent their time. Only 6 years later, because of multiple changes in the federal and city regulations, low-fat and skim are the norm for NYC children older than 2 years. It is likely that reversing the childhood obesity epidemic will similarly require layering of multiple policy, system, and environmental changes to bring levels down to those of past decades.

We hope that colleagues from other jurisdictions, operators of early childhood services, and national policy makers will find this evaluation useful in their efforts to design and implement programs and policies to address childhood obesity for their communities.
